# Solitary right hepatic artery passing anterior to the pancreas: a rare and challenging anatomical variant during Whipple procedure

**DOI:** 10.1093/jscr/rjag312

**Published:** 2026-04-28

**Authors:** Hazem Alouani, Annouar Oueslati, Salsabil Nasri, Raja Boussassi, Fares Ben Saad, Hichem Jerraya, Ramzi Nouira

**Affiliations:** Department of General Surgery B, Charles Nicolle Hospital, Tunis, Tunisia; Department of General Surgery B, Charles Nicolle Hospital, Tunis, Tunisia; Faculté de Médecine de Tunis, Université Tunis el Manar, Boulevard Avril 1938, 1006, Tunis, Tunisia; Department of General Surgery B, Charles Nicolle Hospital, Tunis, Tunisia; Faculté de Médecine de Tunis, Université Tunis el Manar, Boulevard Avril 1938, 1006, Tunis, Tunisia; Department of Anesthesiology and Critical Care, Charles Nicolle Hospital Tunis, Tunisia; Department of General Surgery B, Charles Nicolle Hospital, Tunis, Tunisia; Department of General Surgery B, Charles Nicolle Hospital, Tunis, Tunisia; Faculté de Médecine de Tunis, Université Tunis el Manar, Boulevard Avril 1938, 1006, Tunis, Tunisia; Department of General Surgery B, Charles Nicolle Hospital, Tunis, Tunisia; Faculté de Médecine de Tunis, Université Tunis el Manar, Boulevard Avril 1938, 1006, Tunis, Tunisia

**Keywords:** hepatic artery variation, superior mesenteric artery, pancreaticoduodenectomy, Whipple, vascular reconstruction

## Abstract

Anomalous hepatic arterial anatomy is a critical consideration during pancreaticoduodenectomy. We report a rare case of a solitary right hepatic artery (RHA) arising from the superior mesenteric artery with complete absence of the common hepatic artery, coursing through the pancreatic parenchyma and supplying the entire liver. A 62-year-old man underwent a Whipple procedure for a pancreatic head tumour. Intraoperatively, the RHA was preserved and reconstructed using an interposition graft from the great saphenous vein to maintain hepatic perfusion. The postoperative course was uncomplicated, and the patient was discharged on postoperative Day 9. Awareness of such variants is essential to prevent catastrophic hepatic ischemia during pancreatic surgery.

## Introduction

Pancreaticoduodenectomy requires meticulous knowledge of arterial anatomy to avoid vascular injury and postoperative hepatic ischemia. Variations in hepatic arterial supply occur in up to 40% of individuals, most commonly involving accessory or replaced branches arising from the superior mesenteric artery (SMA) [1]. However, the complete absence of the common hepatic artery with a solitary right hepatic artery (RHA) originating from the SMA and traversing the pancreatic head is exceptionally rare and poses major operative challenges [2].

Preservation or reconstruction of such an artery is crucial when performing oncological resection of pancreatic head lesions. Here, we describe a unique case of a solitary SMA-origin RHA coursing intraparenchymally through the pancreas, requiring vascular reconstruction during Whipple procedure to maintain hepatic arterial flow.

## Case report

**Figure 1:**
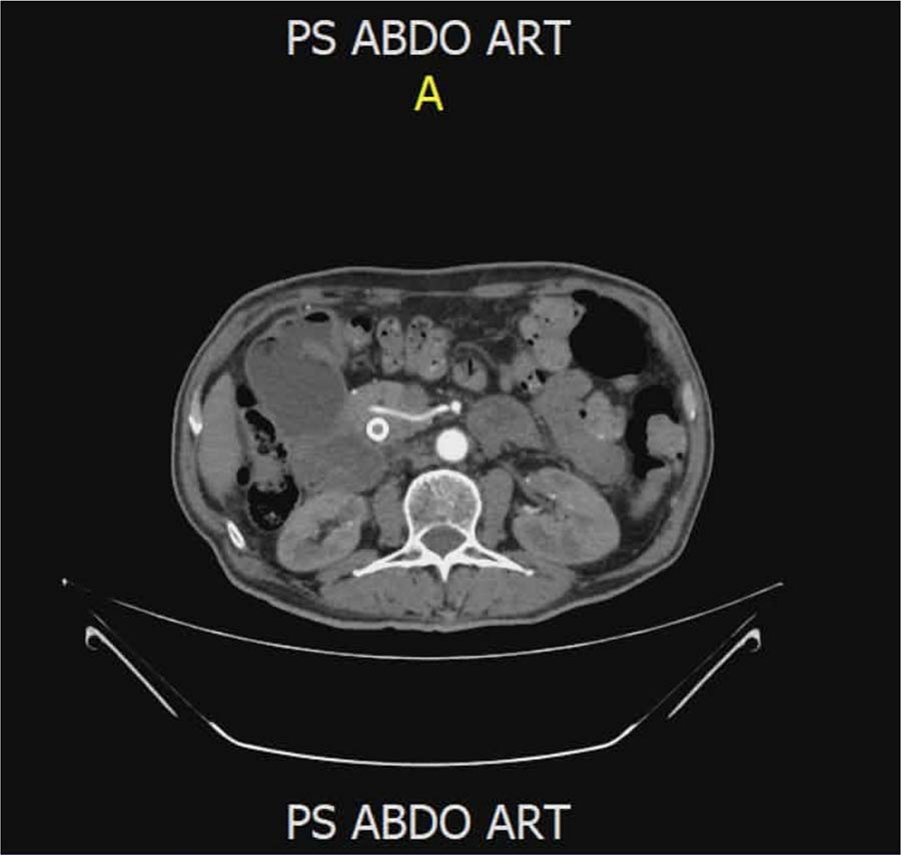
Preoperative CT scan showing the solitary right hepatic artery arising from the SMA and coursing through the pancreatic parenchyma.

**Figure 2:**
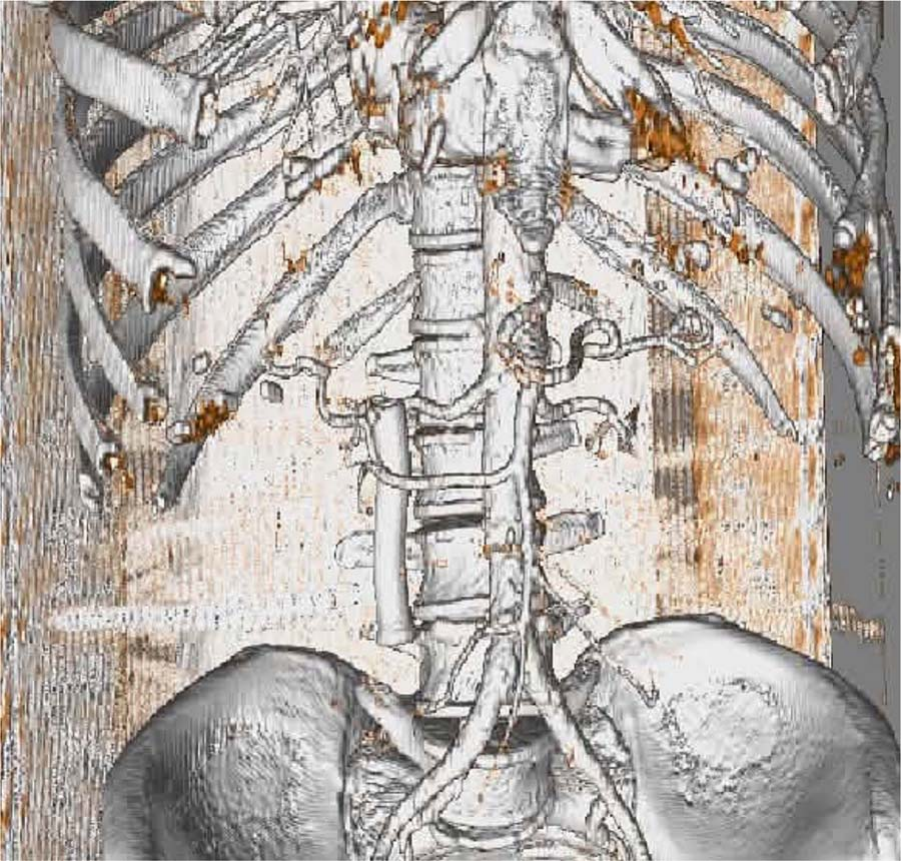
3D CT angiography reconstruction showing the solitary RHA arising from the SMA and coursing through the pancreatic parenchyma. This reconstruction confirms that the RHA is the sole arterial supply to the liver.

A 62-year-old man presented with jaundice and abdominal discomfort. Laboratory tests showed cholestatic liver enzyme elevation. Contrast-enhanced computed tomography (CT) revealed a mass in the pancreatic head consistent with a resectable neoplasm. Preoperative imaging demonstrated an unusual arterial anatomy: a single RHA arising directly from the SMA, with no identifiable common hepatic artery, and the artery was seen passing through the pancreatic parenchyma before ascending toward the hepatic hilum (Fig. 1). A 3D CT angiography reconstruction (Fig. 2) clearly demonstrates the entire course of this solitary RHA and confirms that it represents the exclusive arterial supply to the liver.

**Figure 3:**
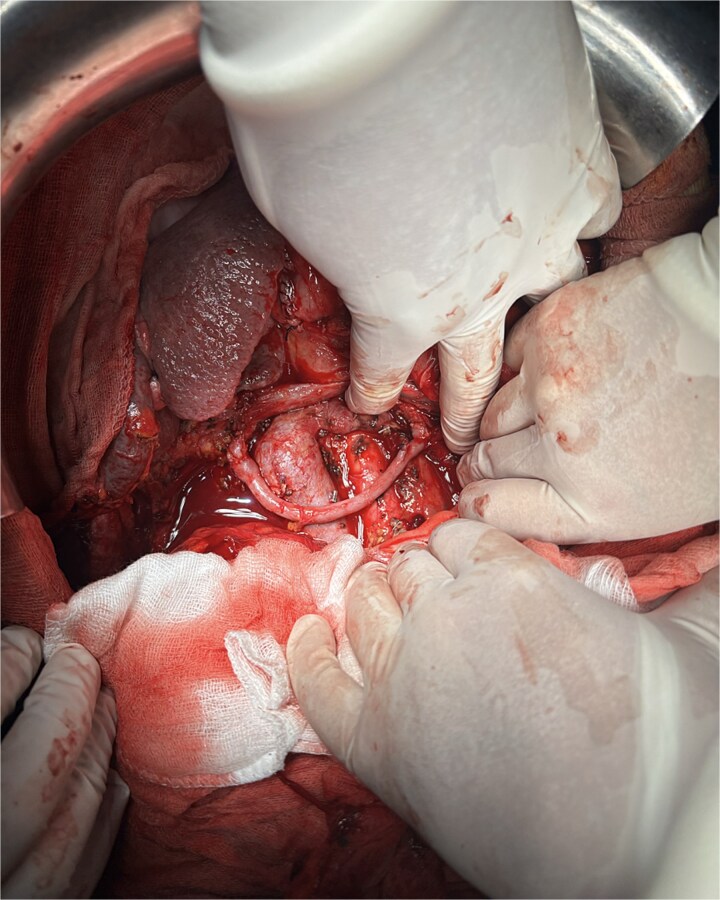
Intraoperative photograph showing the isolated aberrant artery before reconstruction.

**Figure 4:**
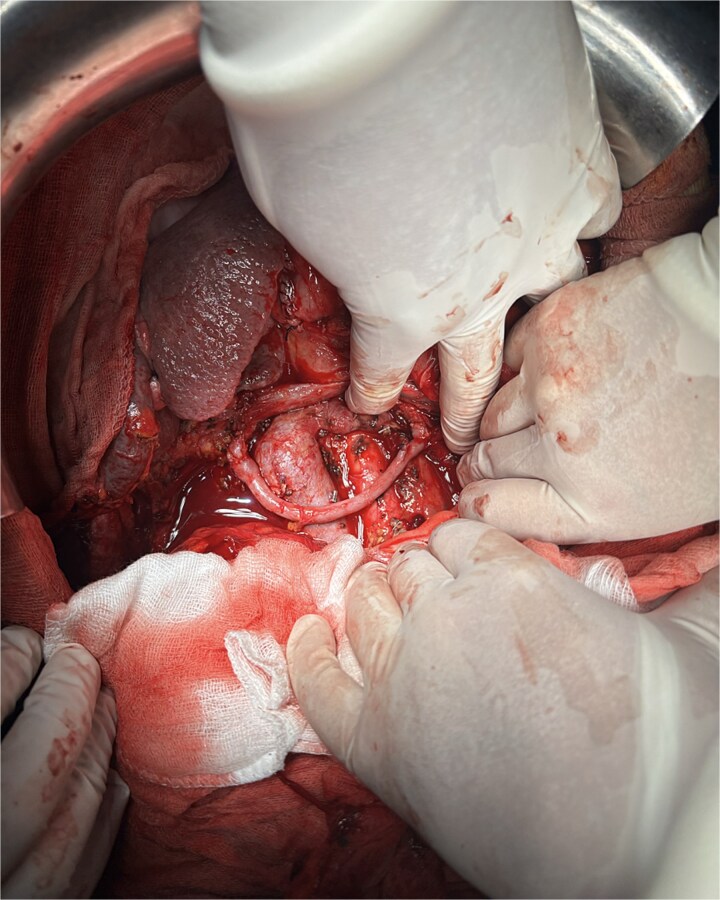
Saphenous vein interposition graft restoring arterial continuity between SMA and RHA.

Intraoperatively, the aberrant artery was confirmed as the sole arterial supply to the entire liver. Dissection around the pancreatic head revealed the artery coursing deeply within the tumouric region (Fig. 3), making preservation without resection impossible. After assessing backflow and hepatic perfusion, the artery was transected, and an interposition graft from the great saphenous vein was fashioned to restore continuity between the SMA and the proximal RHA stump (Fig. 4). Arterial flow was verified with Doppler assessment.

The pancreaticoduodenectomy proceeded uneventfully, with standard biliary and pancreatic reconstruction. Total operative time was 6 hours.

Postoperatively, the patient remained hemodynamically stable with no evidence of hepatic dysfunction. Liver enzymes normalized progressively, and Doppler ultrasound confirmed graft patency. The patient resumed oral intake on postoperative Day 4 and was discharged on Day 9 with no complications.

## Discussion

Hepatic arterial variations occur in up to 45% of individuals, but complete absence of the common hepatic artery with a solitary SMA-origin RHA is exceedingly rare [1]. Most replaced RHAs course posterior to the pancreas, making a transpancreatic trajectory exceptionally challenging [3].

During pancreaticoduodenectomy, the presence of such an artery may significantly increase the risk of unintended vascular injury, postoperative ischemic hepatitis, biliary anastomotic breakdown, and mortality [4].

Preoperative CT angiography, ideally with 3D reconstruction, is essential to identify such variants, guide surgical planning, and anticipate the need for vascular reconstruction, and choice of resection strategy [5]. In the present case, the artery was not only aberrant but also the exclusive arterial inflow to the liver, making its preservation or reconstruction mandatory for safe surgery.

When a solitary RHA traverses the pancreatic head, options include meticulous dissection with preservation, or resection with interposition grafting. Intraoperative Doppler assessment is crucial to confirm liver perfusion. Using autologous grafts such as the saphenous vein provides adequate length and calibre while minimizing tension. Surgeons should be aware of variants outside Michels and Hiatt classifications, as failure to recognize a transpancreatic solitary RHA can result in catastrophic hepatic ischemia [6]. Interposition grafting is often preferred when the tumour’s position mandates arterial sacrifice or when tension-free anastomosis is not possible. Using the saphenous vein provides adequate length, appropriate calibre, and favorable patency in low-pressure hepatic circulation [7]. In this case, graft reconstruction allowed achievement of oncologic clearance while maintaining robust hepatic perfusion.

The uneventful postoperative course and discharge by day nine demonstrate that major vascular reconstruction during PD can be safe and effective when pre-operative vascular anatomy is fully assessed and technical precision is ensured [8]. This case also emphasizes the need for surgeons to consider rare arterial variants outside the classical Michels and Hiatt classifications, as failure to recognize a transpancreatic solitary hepatic artery can lead to catastrophic hepatic ischemia.

## Conclusion

A solitary RHA arising from the SMA and coursing through the pancreas represents a rare but surgically critical arterial variant. When encountered in the setting of pancreatic head malignancy, pancreaticoduodenectomy with arterial reconstruction is feasible and may offer favorable outcomes. Recognition of this variant preoperatively and careful intraoperative management are key to safe pancreaticoduodenectomy. Interposition grafting allows both oncologic clearance and preservation of hepatic perfusion in challenging cases.
